# MTL-CEBPA Combined with Immunotherapy or RFA Enhances Immunological Anti-Tumor Response in Preclinical Models

**DOI:** 10.3390/ijms22179168

**Published:** 2021-08-25

**Authors:** Kai-Wen Huang, Choon Ping Tan, Vikash Reebye, Cheng Ean Chee, Dimitris Zacharoulis, Robert Habib, David C. Blakey, John J. Rossi, Nagy Habib, Mikael H. Sodergren

**Affiliations:** 1 Hepatitis Research Center, Department of Surgery, National Taiwan University Hospital, College of Medicine, Taipei 100, Taiwan; 2 Centre of Mini-Invasive Interventional Oncology, National Taiwan University Hospital, Taipei 100, Taiwan; 3 MiNA Therapeutics Ltd., London W12 0BZ, UK; tan@minatx.com (C.P.T.); v.reebye@imperial.ac.uk (V.R.); robert@minatx.com (R.H.); david.blakey@minatx.com (D.C.B.); 4 Department of Surgery and Cancer, Imperial College London, London W12 0NN, UK; m.sodergren@imperial.ac.uk; 5 Department of Haemato-Oncology, National University Cancer Institute, Singapore 119077, Singapore; cheng_ean_chee@nuhs.edu.sg; 6 Department of General Surgery, University Hospital of Larissa, 41110 Larissa, Greece; zachadim@yahoo.com; 7 Department of Molecular and Cellular Biology, Beckman Research Institute of City of Hope, Duarte, CA 91010, USA; jrossi@coh.org

**Keywords:** CEBPA, abscopal, immunotherapy, PD-1

## Abstract

The transcription factor CEBPA is a master regulator of liver homeostasis, myeloid cell differentiation and is downregulated in several oncogenic diseases. MTL-CEBPA is a small activating RNA drug which upregulates gene expression of CEBPA for treatment of hepatocellular carcinoma (HCC). We investigate whether MTL-CEBPA has immune modulatory effects by combining MTL-CEBPA with an anti-PD-1 checkpoint inhibitor (CPI) and/or radiofrequency ablation (RFA) in two preclinical models. First, mice with two flanks of HCC tumors (BNL) were treated with combinations of RFA (right flank), anti-PD-1 or MTL-CEBPA. The reduction of the left flank tumors was most pronounced in the group treated with RFA+anti-PD1+MTL-CEBPA and 7/8 animals responded. This was the only group with a significant increase in CD8+ and CD49b+/CD45+ tumor infiltrating lymphocytes (TIL). Second, a combination of anti-PD-1+MTL-CEBPA was tested in a CT26 colon cancer model and this treatment significantly reduced tumor size, modulated the tumor immune microenvironment and increased TILs. These data suggest a clinical role for combination treatment with CPIs, RFA and MTL-CEBPA through synergistic priming of the immune tumor response, enabling RFA and CPIs to have a pronounced anti-tumor effect including activity in non-treated tumors in the case of RFA.

## 1. Introduction

The transcription factor CEBPA (CCAAT/enhancer-binding protein alpha) is a leucine zipper protein which acts as a master regulator of liver homeostasis, including albumin production, and multiple oncogenic processes including cell cycle control, proliferation and angiogenesis. It is also a master regulator of the hematopoietic myeloid cell lineage, in which it primes and activates the myeloid gene expression program by binding to promoters or enhancers of myeloid-related genes [1,2]. Myeloid lineage specific genetic ablation of CEBPA has been shown to promote tumor growth by increasing the number and tumor infiltration of myeloid-derived suppressor cells (MDSC) leading to a pro-angiogenic, immune suppressive and pro-tumorigenic environment [3]. Furthermore, CEBPA protein is found to be downregulated in the peritoneal macrophages of tumor-bearing mice [4]. MTL-CEBPA comprises a double stranded RNA payload formulated inside a SMARTICLES® liposomal nanoparticle to specifically target and upregulate the CEBPA gene [5]. In a hepatocellular carcinoma (HCC) cell line transfected with MTL-CEBPA, increased levels of both CEBPA and albumin mRNA in addition to a 3-fold increase in albumin secretion and 50% decrease in cell proliferation was observed [6,7]. In a cirrhotic rat model with multifocal HCC, the i.v. injection of a formulated small activating RNA (saRNA) to CEBPA decreased the tumor burden by 80%, as well as increasing circulating albumin up to 30% [8,9,10]. MTL-CEBPA is the first saRNA and the first drug targeting CEBPA to enter phase 1 clinical trials, where it has been shown to be associated with limited toxicity and has shown promising initial clinical response in patients with advanced HCC [11,12]. The impact on circulating white blood cell populations suggest that the mechanism may be through a significant immuno-modulatory effect on the tumor immune microenvironment (TIME) through an impact on myeloid cells.

The PD-1 inhibitor Nivolumab, which causes the activation of T-cells and cell-mediated immune responses against tumor cells, has shown clinical activity in a range of tumors. It gained accelerated FDA approval for second-line treatment of HCC based on a subgroup of the CHECKMATE-040 trial [13]. Patients treated with Nivolumab showed an overall response rate of 14.3% (95% CI: 9.2, 20.8), with 3 complete responses and 19 partial responses. Response duration ranged from 3.2 to 38.2+ months; 91% of responders had responses lasting 6 months or longer and 55% had responses lasting 12 months or longer.

Radiofrequency ablation (RFA) is the process by which a tumor is destroyed using heat generated by a high frequency alternating current and applied through an electrode tip. RFA is one of the standard treatment options for HCC in clinical practice and is associated with a significant survival benefit [14]. Following RFA, the localized coagulation necrosis of the tumor remains in the body and provides proinflammatory signals to induce the release of large amounts of cellular debris, a source of tumor antigens, which can trigger a host-adaptive immune response against the tumor [15]. Evidence suggests that tumor thermal ablation induces modulation of both innate and adaptive immune systems, inducing anti-tumor immune responses through efficient loading of dendritic cells, enhanced antigen presentation and an amplified tumor-specific T-cell response [16,17,18,19,20].

We hypothesize that the oncological efficacy of MTL-CEBPA may be enhanced by combination treatment with either PD-1 inhibition, RFA therapy or a combination of RFA and PD-1 through synergism of immuno-modulatory response. The aim of this study was to evaluate the therapeutic response of combination therapy of MTL-CEBPA and anti-PD-1 therapy with or without RFA in two distinct mouse syngeneic cancer models and to characterize changes in the immune infiltrate following treatment.

## 2. Materials and Methods

### 2.1. Mice

For the BNL model, BALB/c mice were purchased from BioLasco Co. (Taipei, Taiwan) and animal studies were performed in compliance with approval from the Institutional Animal Care and Use Committee of College of Medicine, National Taiwan University. For the CT26 anti-tumor study carried out by Alderley Park Ltd, Alderley Park, Macclesfield, Cheshire, UK under UK Home Office legislation. Female Balb/c mice (6 weeks old) were purchased from Envigo UK.

### 2.2. Tumor Cell Lines

BALB/c-derived murine hepatocellular carcinoma cell line BNL 1ME A.7R.1 (BNL; ATCC, Manassas, VA, USA) was cultured in DMEM supplemented with 10% fetal bovine serum (FBS) and antibiotics (penicillin 100 units/mL, streptomycin 100 μg/mL and amphotericin 25 μg/mL) (Gibco BRL, Gaithersburg, MD, USA). The murine colon tumor cell line CT26.WT (CRL-2638) was grown in RPMI-1640 media with 10% FCS and 2 mM glutamine at 37 °C and 5% CO_2_.

### 2.3. BNL Model Experimental Groups and Treatment Schedule

64 male BABL/c mice were implanted in bilateral flanks by subcutaneous (s.c.) injection of 50 μL of BNL cell suspension containing 5 × 10^5^ cells. Mice were randomly allocated to one of the following 8 experimental groups (8 animals/group):

1. Control
2. RFA
3. Anti-PD1
4. MTL-CEBPA
5. Anti-PD1+ MTL-CEBPA
6. RFA + anti-PD1
7. RFA+ MTL-CEBPA
8. RFA+ Anti-PD1+ MTL-CEBPA

Four weeks after cancer-cell injection, when the tumor reached the diameter of ~1.5 × 1.5 cm, one of the bilateral tumors was treated by RFA at day 0. MTL-CEBPA (3 mg/kg) was given by i.v. injection on days 0, 2 and 5 post RFA treatment. Anti-PD-1 (RMP1-14, BioXCell, West Lebanon, NH, USA), at 200 μg/mouse/dose, was given intraperitoneally (i.p.) on days 0, 2 and 5 post RFA treatment. The tumor sizes were assessed using microcalipers, and the tumor volumes were calculated using the following equation: volume = length × (width)^2^ × 0.5. Mice were sacrificed on day 7.

### 2.4. Radiofrequency Ablation (RFA) Treatment

Animals were anaesthetized with (i.p.) injection of Ketamine/Xylazine solution and positioned prone. After shaving the area, a 22-gauge needle with a 4-mm active tip electrode and EUS-radiofrequency (RF) ablation system (Rita) was used for energy delivery by inserting it into the right flank tumor. A 500-kHz RF generator was used to maintain an output of 10 W. Treatment varied from 1 to 3 min depending on the tumor volume, with all tumors having similar RFA outcome as determined by tissue impedance at the end of the procedure. Seven days after RFA, mice were scarified with collection of the left flank tumor and spleen to prepare tumor-infiltrating lymphocytes for further analysis. Peripheral blood samples were obtained in heparin-containing tubes before and after treatment.

### 2.5. TILs Isolation

To prepare TILs, tumors were harvested and dissected into approximately 5 mm fragments followed by agitation in 0.05 mg/mL collagenase IV and 0.01 mg/mL DNase I in RPMI medium at 37 °C for 40 min. Tumors were minced and filtered through 70-μm and 40-μm nylon mesh to remove debris. Cells were then separated on a Ficoll-Hypaque gradient and used for further analysis.

### 2.6. Flow Cytometry

Tumor tissues were processed, brought to single cell suspensions in PBS with 0.5% BSA and stained at 4 ℃ for 30 min. The cell surface markers were stained with fluorescent-labelled antibodies: FITC-CD45, PE-CD8, PerCP-CD3, CD49b and APC.Cy7-CD4 from BD Biosciences (San Jose, CA, USA). Cells were then washed twice and fixed with a buffer (BD Biosciences, San Jose, CA, USA). The total number of individual leukocyte subsets was determined using 123 count eBeads counting beads (eBioscience, San Diego, CA, USA). Flow cytometry was performed by FACSVerse^TM^ (Becton Dickinson, Mountain View, CA, USA) and the data were processed using FlowJo^TM^ software (Ashland, Oh, USA).

### 2.7. CT26 Model Experimental Group and Treatment Schedule

Balb/c mice were transplanted s.c. with 0.1ml of CT26.WT at 5 × 10^5^ cells per mouse and the order of the cell implant was randomized by box and were allocated to one of the following 4 experimental groups (10 animals/group):

1. Control group
2. MTL-CEBPA
3. Anti-PD1
4. Anti-PD1+ MTL-CEBPA

Dosing commenced on day 1 post cell implant and MTL-CEBPA (5 mg/kg) was given by i.v. injection twice weekly on day 1 and day 3. Anti-PD-1 (RMP1-14, BioXCell, 2bscientific, Upper Heyford, UK), at 10 mg/kg, was given i.p. twice weekly on day 1 and day 4. Animals received a total of 7 doses at the end of the experiment and the control group received phosphate buffered saline (PBS). The tumor sizes were assessed using microcalipers 3 times weekly and the tumor volumes were calculated using the elliptical formula (pi/6 × width × width × length). Mice were humanely killed on day 23 and tumor samples were collected and snap frozen for gene expression analysis.

### 2.8. Nanostring Analysis of CT26 Frozen Tumor Samples

A total of 20 tumor samples were used for nanostring analysis, the control and anti-PD-1 treated group had 4 samples each and the MTL-CEBPA and anti-PD-1+MTL-CEBPA had 6 samples each. Tumor samples were homogenized in QIAzol lysis reagent (Qiagen), 1-Bromo-3-chloropropane (Sigma, St. Louis, MO, USA) was added to each sample and vortexed followed by centrifugation in a pre-chilled centrifuge at 4 °C. The aqueous upper phase was then transferred into a centrifuge tube containing ethanol and the samples were mixed by gentle pipetting. The samples were then transferred into RNeasy columns (Qiagen 74106) and RNA extraction was performed according to the instructions in the kit and RNA was quantified using the QIAxpert system. Nanostring analysis was carried out using a nanostring machine and using nanostring mouse 360 IO codeset (LBL-10545-01) and mouse myeloid innate immunity codeset (LBL-10398-02).

### 2.9. Statistics Analysis

Data are presented as mean ± standard deviations. Statistical significance was assessed by the two-tailed unpaired Student’s *t*-test. The differences were considered significant when the *p* value was less than 0.05. GraphPad Prism (GraphPad Software Inc., San Diego, CA, USA) was used for the analyses.

## 3. Results

### 3.1. Combination of RFA, Anti-PD-1 and MTL-CEBPA Synergistically Reduce BNL Tumor Volume

**Figure 1:**
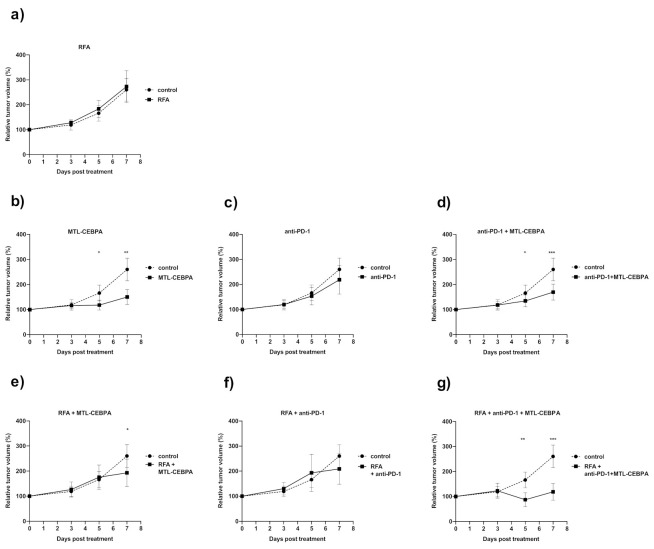
The combination of RFA, anti-PD-1 and MTL-CEBPA synergistically reduce BNL tumor volume. Changes in mean volume of contralateral tumor for (**a**) RFA, (**b**) MTL-CEBPA, (**c**) anti-PD-1 or (**d**) anti-PD-1 + MTL-CEBPA compared to the control group. The following groups were treated with a combination of RFA and (**e**) MTL-CEBPA, (**f**) anti-PD-1 or (**g**) anti-PD-1 + MTL-CEBPA. Statistical significance is indicated as * *p* < 0.05, ** *p* < 0.01 and *** *p* < 0.001.

We investigated whether the combination of RFA, MTL-CEBPA and anti-PD-1 antibody could have enhanced efficacy in the treatment of hepatocellular carcinoma model (BNL 1ME A.7R1). Bilateral flanks of mice were implanted with BNL cells by s.c. injection. When the average tumor size reached the desired volume, half of the animals were treated with RFA on the right flank tumor. Mice from allocated groups were also treated with MTL-CEBPA (i.v.), anti-PD-1 (i.p.) or a combination of both. All animals completed their designated treatment allocation. RFA treatment successfully ablated all right flank tumors and relative tumor volume of non-RFA treated, contralateral left flank tumors were monitored post treatment (Figure 1).

At the end of the experiment, the growth of contralateral tumors in the group only receiving RFA treatment were not significantly different from untreated animals and both groups displayed progressive disease (Figure 1a, Table 1 and Table 2). Mice treated only with MTL-CEBPA retarded the growth of tumors compared with the untreated control (*p* < 0.01) (Figure 1b). Anti-PD-1 only had a small non-significant effect on tumor growth (Figure 1c) and the combination of MTL-CEBPA and anti-PD-1 did not appear to show synergistic effect in the absence of RFA, possibly due to the modest activity of anti-PD-1 in this model (Figure 1d).

In terms of therapeutic responses (Table 1), the combination of RFA with MTL-CEBPA or anti-PD-1 improved the therapeutic response in the contralateral tumor, shifting progressive disease towards stable disease or partial response. The best response was observed in the triple combination group (RFA + anti-PD-1 + MTL-CEBPA) where 7/8 tumors responded, 5/8 animals giving partial response and 2/8 animals with complete response. The latter was not observed in any other treatment group.

**Figure 2:**
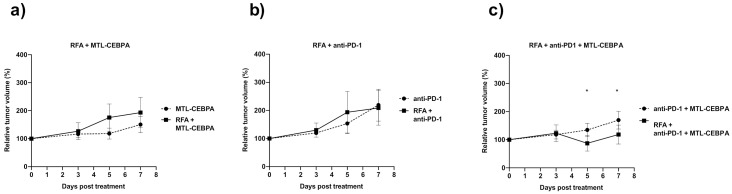
RFA improves the efficacy of anti-PD-1 + MTL-CEBPA combination. Relative tumor volume changes with and without RFA treatment in combination with (**a**) MTL-CEBPA, (**b**) anti-PD-1 or (**c**) anti-PD-1 + MTL-CEBPA. Statistical significance is indicated as * *p* < 0.05.

For endpoint tumor volumes of animals treated in combination with RFA (Figure 1e–g), contralateral tumors of mice treated with RFA + anti-PD-1 + MTL-CEBPA were the smallest among all treatment groups (Figure 1g and Table 2). Mice treated with the combination of RFA + anti-PD-1 + MTL-CEBPA were also the only group that showed significantly smaller tumor volume compared to their non-RFA treated counterpart (Figure 2a–c). This shows the synergistic benefits of applying RFA, anti-PD-1 and MTL-CEBPA in the treatment of mouse hepatocellular carcinoma in this model.

### 3.2. MTL-CEBPA Enhances CD8+ and NKT Cells Infiltrating in BNL Tumor

**Figure 3:**
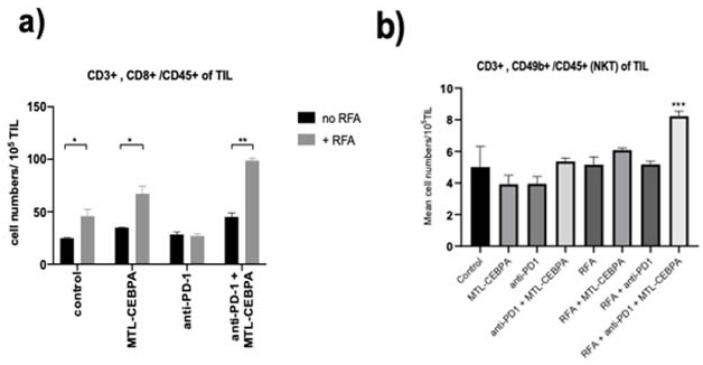
MTL-CEBPA enhances CD8+ and NKT cells infiltrating in BNL tumor. Changes in (**a**) tumor infiltrating cytotoxic T lymphocytes and (**b**) tumor infiltrating Natural Killer T-cells measured by flow cytometery analysis of tumor samples. Statistical significance is indicated as * *p* < 0.05, ** *p* < 0.05, *** *p* < 0.05.

To further explore whether the immune response contributes to the anti-tumor effect, TILs from mice after RFA and/or drug treatment groups were measured by flow cytometry. As can be seen from Figure 3a, the CD8+ T-cell infiltration increased significantly for both the RFA group and the MTL-CEBPA + RFA combination group compared to the control. Although the anti-PD-1 treated group did not show RFA induced CD8+ T-cell infiltration, the combination of anti-PD-1 with MTL-CEBPA shows the largest and most significant increase when mice were also treated with RFA (Table 3 and Figure 3a). This result demonstrates the enhanced anti-tumor immune response when mice were treated with a combination of the three therapies.

NK and NKT lymphocyte count in the tumor was not significantly different among the treatment groups without RFA (Figure 3b). In contrast, we observed an increase of NKT lymphocytes in TILs in MTL-CEBPA, the anti-PD-1 with RFA combination treatment group (Figure 3b).

### 3.3. Combination of Anti-PD-1 and MTL-CEBPA Have Synergistic Efficacy in CT26 Syngeneic Mouse Model

**Figure 4:**
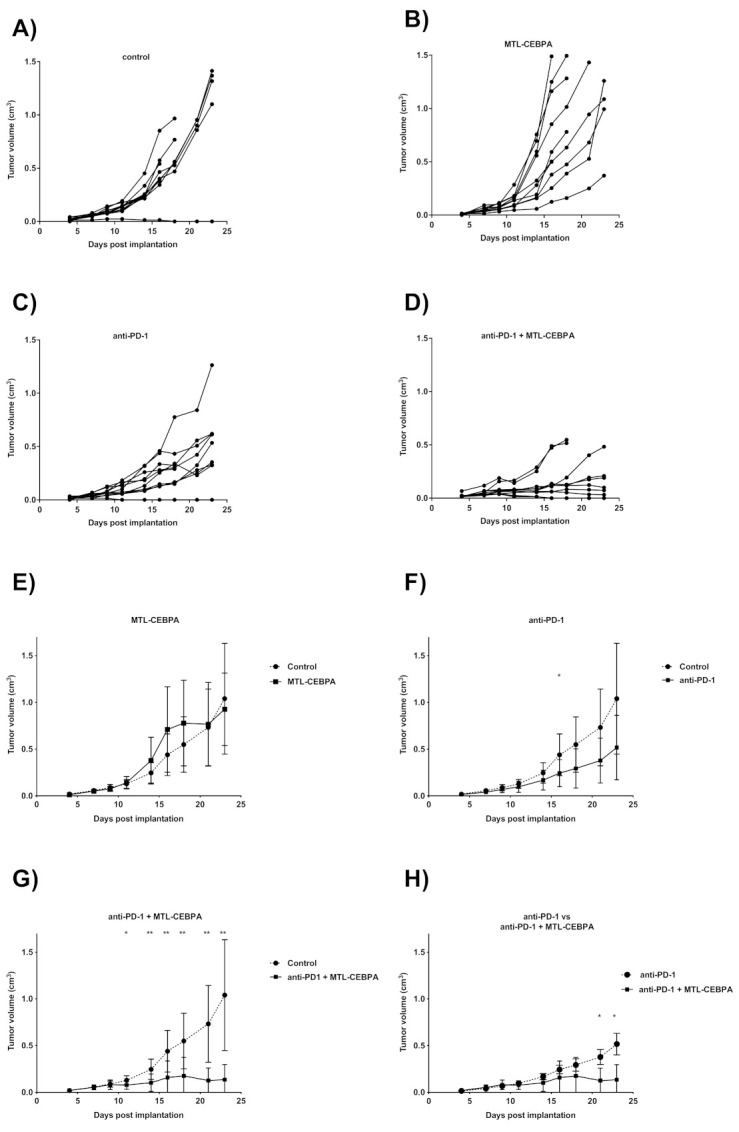
The combination of anti-PD-1 and MTL-CEBPA synergistically reduces CT26 tumor volume. Changes in tumor volume from day 4 to the end of study at day 23. Individual tumor volume for (**A**) control, (**B**) MTL-CEBPA, (**C**) anti-PD-1 and (**D**) anti-PD-1+MTL-CEBPA treated animals were represented. Mean tumor volume of (**E**) MTL-CEBPA, (**F**) anti-PD-1 and (**G**) anti-PD-1+MTL-CEBPA treated animals were compared against the control group (**H**) Comparison of mean tumor volume for anti-PD-1 and anti-PD-1+MTL-CEBPA treated group. Statistical significance is indicated as * *p* < 0.05 and ** *p* < 0.01.

To investigate whether MTL-CEBPA could enhance the efficacy of anti-PD-1 in other tumor models, we tested a combination of MTL-CEBPA and anti-PD-1 in a syngeneic mouse colon tumor model (CT26) which is sensitive to anti-PD-1 treatment. Mice were implanted with CT26 cell lines on one flank and were treated one day later with MTL-CEBPA (i.v.), anti-PD-1 (i.p.) or a combination of both treatments. Palpable tumors were observed from day 4 post implantation and individual tumor volumes were recorded until the end of experiment at day 23 (Figure 4A–D). Mean tumor volumes for each treatment group were compared against the control group (Figure 4E–G) and the final mean tumor volumes for each group are summarized in Table 4.

MTL-CEBPA treated animals had no significant difference in tumor volume compared to the control group at all timepoints (Figure 4B,E) and anti-PD-1 treated animal only showed one time point in the study at which tumor volume was significantly smaller than the control group (Figure 4C,F day 16). In contrast, mean tumor volume of animals treated with anti-PD-1+MTL-CEBBPA were significantly lower than the control group from day 11 onwards (Figure 4G) and individual tumor volume indicated that, at the end of the experiment, 5/8 animals in this group had regressing tumor growth. The remaining animals in this group were also showing slower tumor growth compared to all other groups (Figure 4A–D). Notably, towards the end of the experiment, the anti-PD-1+MTL-CEBBPA group had a significantly smaller tumor volume compared to anti-PD-1 treatment alone (Figure 4H, day 21 and 23). This indicates the synergistic effects of combining anti-PD-1 with MTL-CEBPA in the treatment of CT26 model.

### 3.4. Gene Expression Analysis of CT26 Tumor Reveals That Anti-PD-1+MTL-CEBPA Treated Group Have Anti-Tumor Transcriptional Profile

**Figure 5:**
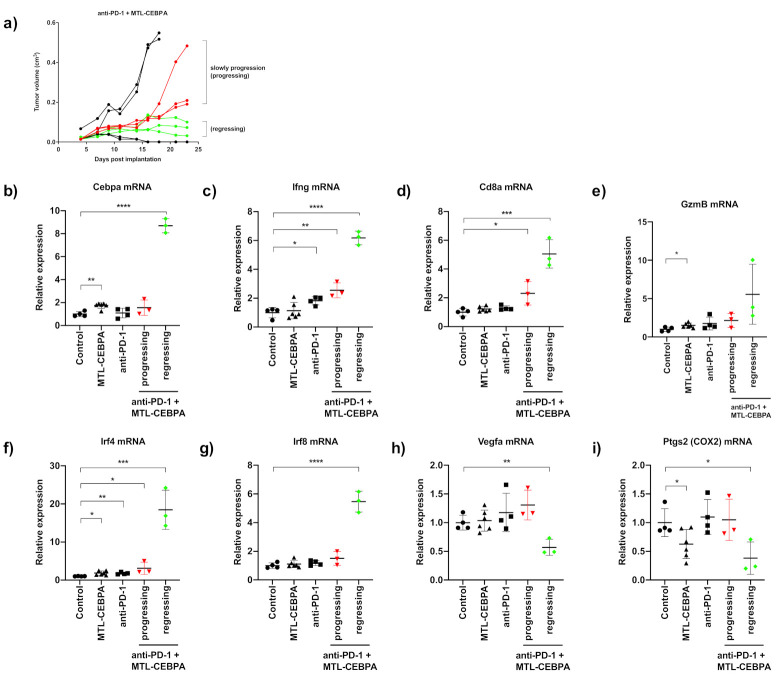
Anti-PD-1+MTL-CEBPA treated mice display anti-tumor transcriptional profile in CT26 tumor (**a**) Segregation of tumor samples in the anti-PD-1+MTL-CEBPA group for nanostring analysis, selected regressing tumors are indicated with the green lines and progressing tumors are indicated with the red lines. Relative expression of (**b**) CEBPA, (**c**) Ifng, (**d**) Cd8a, (**e**) GzmB (**f**) Irf4, (**g**) Irf8, (**h**) Vegfa and (**i**) Ptgs2 mRNA compared to the control group. Statistical significance is indicated as * *p* < 0.05, ** *p* < 0.01, *** *p* < 0.001 and **** *p* < 0.0001.

As half of the tumors in the anti-PD-1 + MTL-CEBPA group were regressing in size towards the end of the experiment, RNA samples from this treatment group were sub-divided into a slowly progressing group and a regressing group for gene expression analysis (Figure 5a). Normalized mRNA expression values derived from nanostring analysis were calculated as fold changes compared to the control group. The MTL-CEBPA treated group had significant upregulation of CEBPA mRNA (1.74 fold, *p* = 0.0012) and the tumor regressing subgroup of anti-PD-1+MTL-CEBPA had a high expression of CEBPA mRNA (8.69 fold, *p* < 0.0001) (Figure 5b). The strong expression of CEBPA mRNA in the regressing group is most likely due to immune activation through the synergistic effects of anti-PD-1 and MTL-CEBPA as all samples in this treatment group displayed increased interferon-gamma (Ifng mRNA) expression (Figure 5c). The regressing group also showed the highest level of Cd8a mRNA and Granzyme B (GzmB) mRNA, suggesting cytotoxic T-cell infiltration and antitumor activity (Figure 5d,e).

For genes related to MDSC biology, we observed significant upregulation of interferon regulatory factor 4 (IRF4) and interferon regulatory factor 8 (IRF8) in the regressing group. Both of these genes have been shown to be downregulated in a MDSC rich environment and their increase in expression correlated with the reversal of the MDSC phenotype (Figure 5f,g) [21,22]. Consistent with the increase in angiogenesis in the tumors as a result of CEBPA deletion described by Mackert et al. [3], RNA extracted from the tumors of MTL-CEBPA + PD1 expressed high levels of CEBPA and showed decreased expression of Vegfa (Figure 5h). Finally, the COX2 gene (Ptgs2 mRNA) that is required for the cancer-promoted induction of MDSC was reduced in MTL-CEBPA treated samples and significantly reduced in MTL-CEBPA +PD-1 treated samples (Figure 5i) [23].

**Figure 6:**
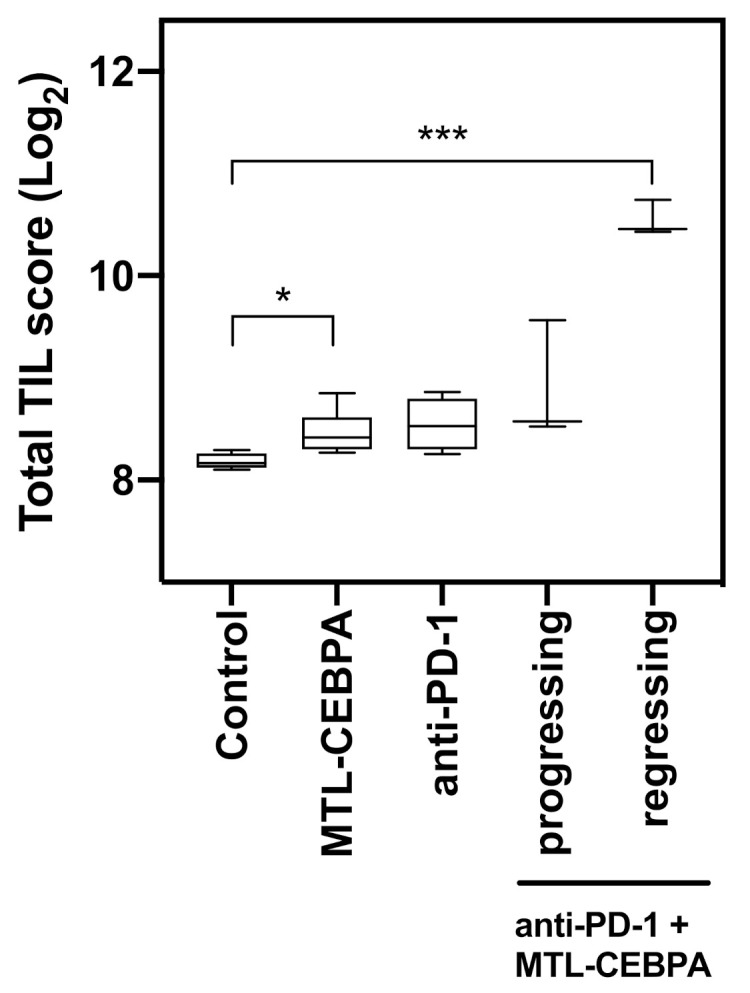
Anti-PD-1+MTL-CEBPA treated mice have increased TIL in CT26 tumor. Tumor infiltrating lymphocyte score for each treatment group analyzed by nanostring analysis of the mouse I/O 360 codeset. Statistical significance is indicated as * *p*< 0.05, *** *p* < 0.001.

Finally, we determined the difference in tumor infiltrating lymphocytes by advance analysis of the Nanostring I/O 360 codeset data. nSolver Advanced analysis (Nanostring systems free software) reveals a significant increase in TILs for the tumor regressing subgroup of the anti-PD-1+MTL-CEBPA treated animals (Figure 6). There were much smaller increases for either MTL-CEBPA or PD-1 treatment alone (Figure 6). This experiment highlights that a combination of MTL-CEBPA with anti-PD-1 has immune modulatory effects to the TIME, resulting in better tumor responses in the treatment of CT26 disease models.

## 4. Discussion

From mechanistic evaluation of HCC patients treated with MTL-CEBPA, we have observed that it induced a significant increase in peripheral granulocytes that was transient after a once weekly dose and re-occurred on repeat dosing. qPCR analysis of these cells showed increased mRNA levels of CEBPA and downregulation of PD-L1, adenosine deaminase and CXCR4, suggesting the potential for an immune modulatory effect on the TIME [12]. This observation, together with the fact that the loss of CEBPA expression causes an increase in the tumor MDSC and immunosuppressive environment [3], led to the hypothesis that the clinical efficacy of MTL-CEBPA may be further augmented by therapies that synergistically influence the TIME to produce an enhanced immune response to the tumor.

MTL-CEBPA treatment reduced the growth of mouse HCC flank tumors compared with the control group, both with and without combination treatment with RFA. However, the greatest therapeutic response was seen in the group treated with a combination of MTL-CEBPA, PD-1 inhibitor and RFA. This was also the only group in which a proportion of the animals exhibited a complete response to treatment. As the tumor evaluation in animals treated with RFA were all on the contralateral side, this suggests that RFA treatment resulted in an abscopal effect, i.e. the regression of distant tumor sites owing to the induction of T-cell responses. PD-1 inhibition on its own or in combination with RFA did not significantly decrease the tumor growth compared to the control in this study. Further work will evaluate whether the prolongation of therapy beyond 7 days further increases the response seen in the combination cohorts.

It has been observed that heat shock protein HSP70 can be detected several hours to days after RF treatment [24]. Together with related molecules released by necrotic cells, this leads to the activation and maturation of dendritic cells and thereby the stimulation of CD4+ and CD8+-effector T-cells directed against the major histocompatibility complex class I– dominant, class I subdominant and class II dominant epitopes. Indeed, in the mouse model, it has previously been observed that tumors partially treated with RFA do not only exhibit this response against the primary tumor, but they also observed an abscopal effect. Furthermore, the animals who exhibited eradication of the primary tumor were able to resist a further challenge with tumor implant, implying a durable immunological memory response [18]. den Brok and colleagues elegantly demonstrated that a significantly larger proportion of draining lymph nodes contained tumor antigens following RFA than using a vaccination. Furthermore they showed that in situ tumor ablation can be combined with immunomodulation via checkpoint inhibition (anti-CTLA-4) or regulatory T-cell depletion to enhance the CD8+ T-cell activation and clinical response [17]. Interestingly, in a neoadjuvant murine model, improved survival and antitumor systemic immunity was seen in pre-resectional RFA treatment, supporting its use in cancers associated with a high risk of local or systemic recurrence [25].

In a clinical study of RFA for HCC, Zerbini and colleagues demonstrated RFA-induced or enhanced T-cell responses specific for HCC-associated antigens. They detected a significant increase of patients responsive either to tumor antigens derived from both the untreated hepatocellular carcinoma tissue and the necrotic tumor. Phenotypic analysis of circulating T and natural killer cells showed an increased expression of activation and cytotoxic surface markers. Tumor-specific T-cell responses were not associated with protection from hepatocellular carcinoma relapse, and they concluded that this effect may represent the basis for the development of an adjuvant immunotherapy for patients undergoing RFA treatment for HCC [19].

In this study we observed a significant increase in the tumor associated cytotoxic and natural killer T lymphocytes in the contralateral tumor following treatment with RFA in combination with MTL-CEBPA and PD-1 inhibition. In a clinical trial of non-small cell lung cancer (NSCLC), Schneider and colleagues demonstrated, for the first time, a local and systemic immune response subsequent to RFA, and then complete surgical resection in patients. Along the perimeter of the RFA-treated tumor tissue they found intense infiltrations of CD4+ and CD8+ lymphocytes following resection. In the peripheral blood, the frequency of proinflammatory and immunostimulatory dendritic cells increased after RFA and in T-cell assays a significant increase in T-cell proliferation was detected after RFA and tumor resection. They concluded that the treatment of NSCLC patients with RFA and surgery leads to an activated and highly T-cell-stimulatory phenotype of dendritic cell and that this activation may promote long-term immunity [26]. These corroborative data support the synergistic mechanism seen with MTL-CEBPA and RFA observed in this study.

We did not detect a significant therapeutic response to PD-1 inhibition in this study however there was a clear benefit when this was combined with CEBPA and RFA. Indeed, combining RFA with a CTLA-4 inhibitor has demonstrated clinical activity in a phase 1 trial of hepatocellular carcinoma with a clear increase in CD8+ T-cells in patients who showed a clinical benefit [27].

In work using melanoma and colon cancer cell lines, as well as clinical data from colorectal liver metastases, it has been shown that both the number of TILs and the expression of PD-L1 were increased in the primary tumor following RFA treatment of colorectal liver metastases. Infiltrating T-cells in the distant tumor displayed a potent but transient antitumor effector function, which waned as the tumor regained its growth. They found that PD-L1 expression increased in tumor associated dendritic cells, myeloid-derived suppressor cells and macrophages following RFA, offering a mechanism by which the tumor can evade increased T-cell infiltration and adaptive immune response [28]. This also offers a potential mechanism by which MTL-CEBPA enhances the clinical efficacy of PD-1 inhibition, through priming of macrophages in the TIME.

In the mouse colon CT26 syngeneic model, monotherapy of PD-1 inhibitor or MTL-CEBPA did not show significant reduction of CT26 tumor volume at the end of the study, although PD-1 had some activity early on during treatment. However, a combination of both treatments yields a much greater response in the animals and the majority of tumors in the combination group were regressing in size by the end of experiment. Nanostring analysis demonstrated signatures associated with immune gene activation, an increase of MDSC suppressing genes (IRF4 and IRF8) [21,22], the reduction of MDSC promoting gene (COX2) [23] and the reduction of the angiogenic gene (Vegfa) [3]. Consistent with the BNL HCC model, the anti-PD-1+MTL-CEBPA groups demonstrated a much larger increase in TILs than either anti-PD-1 or MTL-CEBPA only, highlighting the synergistic effects of MTL-CEBPA with PD-1 inhibitor on T-cell activity.

In summary, we have found that MTL-CEBPA enhances the anti-tumor response of radiofrequency ablation and PD-1 inhibition in a pre-clinical HCC model and PD-1 inhibition in a pre-clinical colon cancer model. In both tumor models the enhanced response was associated with increased activated T-cell supporting an activity of MTL-CEBPA on TIME. These data suggest a clinical role for combination treatment with checkpoint blockade and MTL-CEBPA, with or without RFA, through synergistic priming of the immune tumor response.

## Figures and Tables

**Table 1:** Therapeutic response to contralateral flank tumor.

Group	CR	PR	SD	PD
control	0	0	0	8 (100%)
RFA	0	0	0	8 (100%)
anti-PD1	0	0	3 (33.3%)	6 (66.7%)
MTL-CEBPA	0	3 (33.3%)	3 (33.3%)	3 (33.3%)
anti-PD1 + MTL-CEBPA	0	2 (22.2%)	4 (44.4%)	3 (33.3%)
RFA + anti-PD1	0	0	5 (50%)	5 (50%)
RFA + MTL-CEBPA	0	4 (50%)	2 (25%)	2 (25%)
RFA + anti-PD1 + MTL-CEBPA	2 (25%)	5 (62.5%)	1 (12.5%)	0

CR—complete response, PR—partial response, SD—stable disease, PD—progressive disease.

**Table 2:** Illustrates the mean change in tumor volume in the experimental groups.

Group	Mean Volume Change over Starting Size (%) +/− SD	*p* vs. Control
control	260.4 ± 45.4	-
MTL-CEBPA	150.5 ± 29.4	0.001
anti-PD1	219.1 ± 57.1	n.s
anti-PD1 + MTL-CEBPA	169.8 ± 31.6	0.0005
RFA	273.1 ± 63.8	n.s
RFA + MTL-CEBPA	193.2 ± 54.2	0.029
RFA + anti-PD1	209.0 ± 61.1	n.s
RFA + anti-PD1 + MTL-CEBPA	118.6 ± 33.6	0.0006

**Table 3:** TILs in treatment groups.

Group	Mean +/− SD of Tumor Infiltrating Cells (Cell Numbers/10^5^ TIL)			
	CD4	CD8	NK	NKT
control	93.4 ± 1.59	24.8 ± 5.9	7.83 ± 3.23	5.01 ± 1.30
MTL-CEBPA	104.1 ± 5.42	34.9 ± 2.9	10.9 ± 1.98	3.92 ± 0.56
anti-PD1	93.4 ± 1.59	28.5 ± 2.18	9.33 ± 1.82	3.95 ± 0.45
anti-PD1 + MTL-CEBPA	89.5 ± 6.03	45.2 ± 3.45	17.6 ± 1.50 *	5.36 ± 0.23
RFA	128.3 ± 21.2	45.8 ± 6.57 *	13.5 ± 2.93	5.16 ± 0.50
RFA + MTL-CEBPA	150.2 ± 20.9	67.2 ± 7.19 *	16.0 ± 2.34	6.09 ± 0.13
RFA + anti-PD1	95.3 ± 12.3	26.9 ± 1.96	12.7 ± 0.02	5.18 ± 0.20
RFA + anti-PD1 + MTL-CEBPA	116.2 ± 5.9	98.9 ± 2.23 *	21.2 ± 2.58 *	8.23 ± 0.32 ***

* *p* < 0.05, ***p < 0.005.

**Table 4:** Mean CT26 tumor volume in the experimental groups on day 23.

Group	Mean Tumor Volume (cm^3^)	*p* vs. Control
control	1.040 ± 0.593	-
MTL-CEBPA	0.927 ± 0.388	n.s
anti-PD1	0.517 ± 0.345	n.s
anti-PD1 + MTL-CEBPA	0.136 ± 0.161	0.002

## Data Availability

The data presented in this study are available on request from the corresponding author. The data are not publicly available due to IP generated in MiNA Therapeutics.
